# Supplementary material for: Serum Anti-Mullerian Hormone Levels Might Indicate Premenopausal Endometrial Lesions
Source: Diagnostics (Basel). 2023 Oct 25;13(21):3301. doi: 10.3390/diagnostics13213301 (PMC10650321; doi:10.3390/diagnostics13213301)
Supplement: Supplementary file 1 [file diagnostics-13-03301-s001.zip › diagnostics-2642616-supplementary.pdf]

Supplementary Table S1 Baseline comparison of training sets and validation sets for PCOS

Feature	Train set (n = 1209)	Validation set (n = 153)	*p* value
Menophania (year)	30 (28 - 34)	31 (28 - 34)	0.161
Menstrual period (day)	13 (13 - 14)	13 (13 - 14)	0.824
Gravidity	6 (5 - 7)	6 (5 - 7)	0.952
Parity	1 (0 - 2)	1 (0 - 2)	0.835
BMI (kg/m^2)	0 (0 - 0)	0 (0 - 0)	0.135
EPL	22.41 (20.45 - 24.80)	22.06 (19.92 - 24.46)	0.251
PCOS phenotype	90 (7.4%)	16 (10.5%)	0.190
A			0.229
B	399 (33.0%)	50 (32.7%)	
C	263 (21.8%)	27 (17.6%)	
D	114 (9.4%)	10 (6.5%)	
Underlying disease	433 (35.8%)	66 (43.1%)	
Hypertension			
Diabetes mellitus	38 (3.1%)	8 (5.2%)	0.178
Higher education	34 (2.8%)	8 (5.2%)	0.103
Menstrual regularity	760 (62.9%)	101 (66.0%)	0.446
Dysmenorrhea	385 (31.8%)	44 (28.8%)	0.439
History of sex hormone use	279 (23.1%)	40 (26.1%)	0.399
Family history of cancer	72 (6.0%)	13 (8.5%)	0.221
Smoking history	9 (0.7%)	1 (0.7%)	0.901
AMH (ng/ml)	7.83 (6.04 - 10.30)	7.99 (5.71 - 10.41)	0.707
Basal endocrine			
bLH (IU/L)	7.81 (5.32 - 11.80)	6.96 (4.19 - 11.76)	0.164
bFSH (IU/L)	5.86 (5.03 - 6.85)	5.68 (5.012 - 6.90)	0.372
bE_2 (pmol/L)	109.60 (76.09 - 136.63)	110.00 (82.44 - 139.00)	0.806
bP (nmol/L)	0.93 (0.57 - 1.31)	0.95 (0.56 - 1.24)	0.974
TT (nmol/L)	1.10 (0.80 - 1.50)	1.10 (0.80 - 1.40)	0.792
PRL (ng/ml)	14.50 (10.90 - 19.80)	14.10 (10.40 - 19.50)	0.397
Tumor marker			
CEA (ng/ml)	1.10 (0.70 - 1.60)	1.00 (0.70 - 1.50)	0.988
AFP (ng/ml)	2.20 (1.60 - 3.20)	2.30 (1.60 - 3.20)	0.627
CA-125 (U/ML)	14.30 (10.20 - 20.60)	14.70 (9.60 - 20.80)	0.730
CA-153 (U/ML)	8.10 (5.90 - 11.70)	8.00 (5.80 - 12.75)	0.963
CA-199 (U/ML)	8.90 (5.80 - 13.80)	9.00 (6.03 - 14.15)	0.628

PCOS: polycystic ovary syndrome; EPL: endometrial proliferative lesions; BMI: Body Mass Index; AMH: anti-mullerian hormone; bFSH: basal follicular stimulating hormone; bLH: basal Luteinizing hormone; bE2: basal Estradiol; bP: basal progesterone; TT: total testosterone; PRL: prolactin; CEA: carcinoma embryonic antigen, AFP: alpha-fetoprotein.

Supplementary Table S2 Baseline comparison of training sets and validation sets for non-PCOS

Feature	Train set (n = 5366)	Validation set (n = 736)	*p* value
Menophania (year)	31 (28 - 34)	31 (28 - 34)	0.901
Menstrual period (day)	14 (13 - 14)	14 (13 - 14)	0.739
Gravidity	6 (5 - 7)	6 (5 - 7)	0.813
Parity	1 (0 - 2)	1 (0 - 2)	0.773
BMI (kg/m^2)	0 (0 - 1)	0 (0 - 1)	0.982
EPL	21.22 (19.53 - 23.18)	21.30 (19.31 - 23.23)	0.841
Underlying disease	117 (2.2%)	20 (2.7%)	0.356
Hypertension			
Diabetes mellitus	46 (0.9%)	8 (1.1%)	0.533
Higher education	30 (0.6%)	2 (0.3%)	0.421
Menstrual regularity	3329 (62.0%)	460 (62.5%)	0.809
Dysmenorrhea	4782 (89.1%)	657 (89.3%)	0.903
History of sex hormone use	1511 (28.2%)	196 (26.6%)	0.383
Family history of cancer	303 (5.6%)	37 (5.0%)	0.492
Smoking history	52 (1.0%)	10 (1.4%)	0.323
AMH (ng/ml)	3.25 (2.23 - 4.55)	3.26 (2.25 - 4.56)	0.761
Basal endocrine			
bLH (IU/L)	4.69 (3.54 - 5.94)	4.40 (3.41 - 5.62)	0.091
bFSH (IU/L)	6.45 (5.50 - 7.52)	6.48 (5.23 - 7.41)	0.454
bE_2 (pmol/L)	103.15 (75.63 - 133.00)	106.85 (78.18 - 139.00)	0.425
bP (nmol/L)	1.03 (0.68 - 1.40)	1.02 (0.67 - 1.42)	0.995
TT (nmol/L)	0.70 (0.50 - 1.00)	0.70 (0.50 - 1.00)	0.528
PRL (ng/ml)	15.50 (11.60 - 21.50)	15.70 (11.90 - 21.18)	0.378
Tumor marker			
CEA (ng/ml)	1.10 (0.70 - 1.50)	1.10 (0.70 - 1.50)	0.808
AFP (ng/ml)	2.20 (1.60 - 3.20)	2.20 (1.60 - 3.20)	0.928
CA-125 (U/ML)	15.70 (11.50 - 22.60)	15.90 (11.40 - 22.60)	0.985
CA-153 (U/ML)	8.30 (6.10 - 11.80)	8.40 (6.00 - 11.80)	0.866
CA-199 (U/ML)	9.60 (6.30 - 15.10)	9.60 (6.30 - 14.58)	0.508

PCOS: polycystic ovary syndrome; EPL: endometrial proliferative lesions; BMI: Body Mass Index; AMH: anti-mullerian hormone; bFSH: basal follicular stimulating hormone; bLH:

basal Luteinizing hormone; bE2: basal Estradiol; bP: basal progesterone; TT: total testosterone; PRL: prolactin; CEA: carcinoma embryonic antigen, AFP: alpha-fetoprotein.
